# Applications of Artificial Intelligence for the Prediction and Diagnosis of Cancer Therapy-Related Cardiac Dysfunction in Oncology Patients

**DOI:** 10.3390/cancers17040605

**Published:** 2025-02-11

**Authors:** Isabel G. Scalia, Girish Pathangey, Mahmoud Abdelnabi, Omar H. Ibrahim, Fatmaelzahraa E. Abdelfattah, Milagros Pereyra Pietri, Ramzi Ibrahim, Juan M. Farina, Imon Banerjee, Balaji K. Tamarappoo, Reza Arsanjani, Chadi Ayoub

**Affiliations:** 1 Department of Cardiovascular Diseases, Mayo Clinic, Phoenix, AZ 85054, USA; scalia.isabel@mayo.edu (I.G.S.); abdelnabi.mahmoud@mayo.edu (M.A.); ibrahim.omar3@mayo.edu (O.H.I.); abdelfattah.fatmaelzahraa@mayo.edu (F.E.A.); pereyra.milagros@mayo.edu (M.P.P.); ibrahim.ramzi@mayo.edu (R.I.); farina.juanmaria@mayo.edu (J.M.F.); tamarappoo.balaji@mayo.edu (B.K.T.);; 2 Department of Radiology, Mayo Clinic, Phoenix, AZ 85054, USA; banerjee.imon@mayo.edu

**Keywords:** cardio-oncology, artificial intelligence, echocardiography, computed tomography, cardiac magnetic resonance imaging

## Abstract

Cardiovascular diseases and cancer are the leading causes of morbidity and mortality in modern society. Expanding cancer therapies that have improved prognosis may also be associated with cardiotoxicity, and extended life span after survivorship is associated with the increasing prevalence of cardiovascular disease. As such, the field of cardio-oncology has been rapidly expanding, with an aim to identify cardiotoxicity and cardiac disease early in a patient who is receiving treatment for cancer or is in survivorship. Artificial intelligence is revolutionizing modern medicine with its ability to identify cardiac disease early. This article comprehensively reviews applications of artificial intelligence specifically applied to electrocardiograms, echocardiography, cardiac magnetic resonance imaging, and nuclear imaging to predict cardiac toxicity in the setting of cancer therapies, with a view to reduce early complications and cardiac side effects from cancer therapies such as chemotherapy, radiation therapy, or immunotherapy.

## Simple Summary

Heart disease is common in the general population and can complicate the treatment of cancer, particularly in the setting of chemotherapy and immunotherapy, and in the long term following radiation therapy to the chest. Cardiac investigation with electrocardiography and imaging may help diagnose and predict outcomes of patients with cardiovascular complications from cancer therapy. This is important as cardiac events may lead to worse outcomes and may also result in the withholding or withdrawal of potentially lifesaving cancer treatments. Various artificial intelligence tools applied to these different cardiac tests may help suggest increased risk cardiovascular processes and therefore improve the quality and efficiency of care, and potentially reduce the burden of multiple medical tests and hospital presentations for cancer patients. This review summarizes the current landscape of artificial intelligence in evaluating cancer patients for cardiac complications.

## 1. Introduction

The ever-evolving landscape of novel chemotherapy agents, combined with developments in diagnostic modalities, have improved the prognosis and survivorship of patients with cancer. Despite this, cardiovascular disease remains a substantial burden on the morbidity and mortality of cancer patients, not only during cancer therapy but also in the long term [1,2]. Furthermore, there is an increasing risk of cardiovascular complications associated with new antineoplastic therapies, collectively referred to as cancer therapy-related cardiac dysfunction (CTRCD) [3,4]. CTRCD encompasses a spectrum of cardiac pathologies including but not limited to asymptomatic left ventricular systolic dysfunction (LVSD), overt heart failure (HF), arrhythmias, arterial hypertension, hyperlipidemia and myocarditis [5,6].

Although LVSD more typically occurs with agents such as anthracyclines, tyrosine kinase inhibitors, human epidermal growth factor receptor 2 (HER-2) antagonists, proteasome inhibitors, and radiation, it has been seen to occur with a wide range of anti-neoplastic therapies [7,8,9]. Accelerated coronary artery disease (CAD) may also become an issue in cancer survivors, particularly those who have undergone radiation therapy to the chest. Early detection is essential for minimizing long-term cardiovascular complications in survivors [10].

As such, the growing field of cardio-oncology focuses on improving patient prognosis through both the treatment and prevention of cardiovascular complications [11,12]. Recent guidelines recommend a baseline evaluation of both patient- and cancer therapy-specific risk factors to identify patients at increased risk of CTRCD [5]. Following the commencement of cancer therapy, cardiac imaging forms the cornerstone of CTRCD evaluation, with multiple modalities including echocardiography, magnetic resonance imaging, computed tomography, and nuclear imaging offering both diagnostic and prognostic utility [2].

Parallel to this, advancements in artificial intelligence (AI) models have shown significant promise in the evaluation of cardiovascular diseases, not only for diagnosis but also in prediction, risk-stratification and prognostication. Specifically, there has been a substantial emergence of new and ongoing literature regarding the utility of AI in cardiovascular imaging modalities [13]. Consequently, the role of various AI modalities such as convolutional neural networks and deep learning are rapidly changing the landscape of cardiac evaluation in cancer patients [1]. This review aims to summarize the current literature regarding AI in cardio-oncology, with a focus on cardiac imaging and the evaluation of CTRCD.

## 2. Artificial Intelligence

Although the application of AI within healthcare has gained significant attention in recent years, the development of such models has been a topic of the scientific literature for many decades [14]. In essence, AI involves a computer model or technology acting with human intelligence to learn and develop independently [15,16]. To create a model, first it must be taught with a training dataset, then tested and corrected, and then retested or validated on a subsequent (different and ideally external) dataset. In most medical models, two main approaches are utilized: machine learning and deep learning.

Machine learning models are based on “learning from experience”, typically requiring the input of human feedback [16]. Using large datasets, machine learning algorithms act to find patterns from extracted features and create a model [17]. As more data are added, the algorithm will make adjustments and subsequently extrapolate predictions. Machine learning models can be either supervised or unsupervised; supervised models utilize labeled data with a known outcome, whereas unsupervised models use unlabeled data with an unknown outcome [18,19]. Deep learning is a subcategory of machine learning, which utilizes artificial neural networks with multiple hidden layers of correlation between input data and output data [14]. Inspired by brain neurological networks, deep learning models are trained on smaller volumes of labeled data, which are then applied to larger volumes of unlabeled data. Convolutional neural networks are the most commonly employed deep learning models within medical imaging, allowing for the learning of multiple characteristics simultaneously with minimal human intervention [17,20].

## 3. Electrocardiography

**Figure 1:**
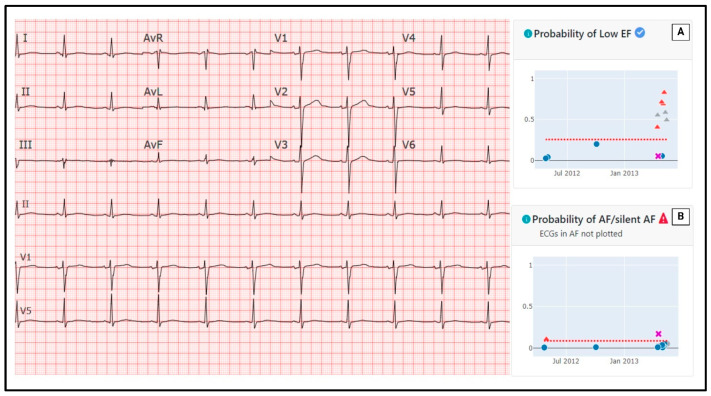
Example of previously validated artificial intelligence models applied to single 12-lead electrocardiogram to predict risk of (**A**) low left ventricular ejection fraction and (**B**) new atrial fibrillation [24,26,27]. Blue dots represent individual ECG recordings with a risk score below the pre-determined cutoff (reflected as the red dashed line), red triangle represent individual ECG recordings with risk scores above the pre-determined cutoff, and purple X represents the currently selected ECG.

A simple 12-lead ECG remains one of the most affordable and accessible diagnostic tools in clinical medicine. The 2022 European Society of Cardiology (ESC) guidelines for cardio-oncology recommend a baseline ECG for all patients prior to cancer therapy (including chemotherapy, immunotherapy or radiation treatment) [5]. Following therapy, the recommended frequency of surveillance ECGs is variable, depending on patient- and cancer therapy-specific risk factors. Despite this, the application of AI algorithms to ECGs has become an increasingly utilized tool for the prediction and prognostication of many conditions in the general population, both cardiac and non-cardiac [21]. AI models have been validated for the prediction of conditions such as atrial fibrillation, aortic stenosis, cardiac amyloidosis, hypertrophic cardiomyopathy, cirrhosis, diastolic dysfunction grade and LVSD, examples demonstrated in Figure 1 [22,23,24,25]. In the instances of structural pathology, AI models are hypothesized to detect subclinical ECG changes that may precede clinically detectable pathology and may not be appreciable by human interpretation [13]. As such, these models hold promise not only for the early detection of pathology but also for potential pre-clinical risk stratification and preventative therapy in cancer patients.

### 3.1. LVSD

Given the frequency and associated morbidity and mortality of CTRCD, several AI models have recently been proposed to assist in the prediction of cancer patients who may develop LVSD, with a focus on patients receiving anthracycline-based chemotherapy. Yagi et al. evaluated the utility of a transfer learning model for the prediction of low LVEF (<40%) in 1011 patients on anthracycline therapy [28]. Using ECGs within 90 days before the commencement of chemotherapy and confirming the diagnosis of CTRCD with TTE (defined as LVEF <53% and >10% drop from baseline), their model had an area under the curve (AUC) accuracy of 0.91 for the detection of CTRCD. Consequently, patients with a high-risk AI score had an adjusted hazard ratio of 2.57 (95%CI 1.62–4.10) for the development of CTRCD at a median follow-up of 560 (IQR 149–999) days.

Oikonomou et al. evaluated a similar ECG-based AI convolutional neural network model on 1308 patients receiving either anthracycline chemotherapy or trastuzumab [29]. Their study again reported a significant correlation between a high-risk AI score and increased risk of CTRCD through a median follow-up of 80 (IQR 42–115) months, with an adjusted hazard ratio of 2.22 (95%CI 1.63–3.02). This study also evaluated 2056 patients receiving immune checkpoint inhibitors; however, no correlation between elevated ECG-AI scores and the incidence of myocarditis was noted.

Similarly, a study by Lee et al. has shown promise in the utility of ECG-AI to predict diastolic dysfunction in the general population [30]. This study developed and validated an ECG-AI model on 98,736 ECGs, with an AUC accuracy of 0.911 for the detection of increased filling pressure, which was seen in higher grades (specifically grades II and II) of diastolic dysfunction. This was further expanded to show accuracy in the diastolic grading of 0.847 (grade ≥ I), 0.911 (grade ≥ II), and 0.943 (grade III). Subsequently, this study reported an increased mortality in patients with ECG-AI-detected increased filling pressure, with an aHR of 1.7 (95%CI 1.7–1.8) over a median follow-up of 5.9 years. Clinically, this may hold significance in the oncology population as diastolic dysfunction may precede systolic dysfunction from therapies such as anthracyclines, trastuzumab, and doxorubicin [7]. These models show promise for the use of ECG-AI as a gatekeeper for further investigation, which may prove important in patients where CTRCD may often be asymptomatic and irreversible when detection is delayed [31].

### 3.2. Arrhythmias

Arrhythmias, particularly atrial fibrillation (AF), are not uncommon in cancer patients receiving antineoplastic therapy and have been specifically associated with agents such as ibrutinib tyrosine kinase inhibitors or in the setting of radiation therapy with mediastinum [21]. Patients with arrhythmias are known to have poorer clinical outcomes, and AF in cancer patients has been associated with higher stroke risk [32,33]. Yet, anticoagulation which mitigates embolic/stroke risk may be associated with a higher bleeding risk in a patient with cancer, and agents such as ibrutinib may be associated with a higher bleeding risk in themselves [34,35].

As such, there is significant clinical potential for AI-ECG to assist in the prediction, early detection, and intervention for cancer patients at risk of new arrhythmias. In the general population, convolutional neural network AI models have been shown to accurately predict the risk of new onset atrial fibrillation from a single sinus rhythm ECG [24,26]. This same AI-ECG model was recently evaluated in a cohort of 754 patients with chronic lymphocytic leukemia, noting patients with a high-risk score (≥0.1) to have a significantly increased risk of new AF (aHR 2.5, 95%CI 1.6–3.9) through a median follow-up of 5.8 years [36]. Similar algorithms have been applied to Holter ECG readings in the general population, which are also important for arrhythmia detection [37]. Multiple convolutional and deep neural networks have been shown to accurately and efficiently detect arrhythmia from Holter ECG recordings [38,39]. These data, along with ongoing developments in transfer learning models, highlight the potential role of previously validated AI technologies to be adapted and validated in specific cohorts such as oncology patients, both for baseline risk stratification and in the setting of potential CTRCD screening.

### 3.3. Cardiac Amyloidosis

Several ECG-AI models have also been evaluated in patients with cardiac amyloidosis [40]. Structural and electrical changes often observed in this disease process offer opportunity for ECG detection including severe conduction disease and arrhythmia, pseudo-infarct patterns, and left ventricular hypertrophy secondary to myocardial infiltration [15]. In 2021, Grogan et al. trained and validated an ECG-AI model on 2997 patients with either light-chain amyloidosis (AL) or transthyretin amyloidosis (ATTR) [41]. This model was found to have an AUC accuracy of 0.91 (95%CI 0.90–0.93) for the detection of cardiac involvement, with a sensitivity of 84% and specificity of 85%. This same group conducted a post-development analysis on the same model, with 440 new amyloid patients and 6600 age-matched controls. Again, the ECG-AI model showed good accuracy for the detection of cardiac involvement (AUC 0.84, 95% CI 0.82–0.86) [42].

Subsequent meta-analyses have reported similar accuracy rates with pooled AUCs of 0.87–0.89 [40,43]. Furthermore, ECG-AI has been shown to have prognostic utility in high-risk patients without a known diagnosis of ATTR or AL, referred for an intervention of severe aortic stenosis [44]. In a cohort of 1426 patients undergoing transcatheter aortic valve replacement (TAVR), those with a high risk of cardiac amyloidosis on ECG-AI (>50% risk score) were found to have an increased risk of mortality (HR 1.40, 95%CI 1.01–1.96) and major adverse cardiac events (HR 1.36, 95%CI 1.13–2.20) through one-year follow-up post TAVR. These studies may suggest further clinical opportunities for the use of ECG-AI, both for diagnosis and prognosis, in other malignancies with infiltrative cardiomyopathy.

### 3.4. Wearables

With the continuous progression of smartwatch and wearable technology, there is increasing potential for a more accessible evaluation of patient ECGs, utilizing only one or two leads. In this setting, Attia et al. reported an accuracy of 0.885 for the detection of low LVEF using a single ECG reading from an Apple watch (Apple Inc. via Apple Health Kit) within 30 days of confirmatory TTE [22]. More recently, Khunte et al. evaluated a similar model in 116,210 patients with paired ECG-TTEs, reporting an AUC accuracy of 0.90 (95%CI 0.89–0.91) in the detection of LVEF < 40%, utilizing a noise-adapted model on Lead I to reflect the input from a smartwatch recording [45]. In parallel, similar modeling has been proposed for the detection of AF using smartwatch technology. The Apple Heart Study evaluated 419,297 patients without a history of AF, reporting a positive predictive value of 0.84 (95%CI 0.76–0.92) for the detection of AF (confirmed by 12-lead ECG) with an irregular pulse notification [46]. In their previously mentioned amyloidosis study, Grogan et al. also reported good accuracy for their model to predict cardiac amyloidosis from both a single-lead (Lead V5 AUC 0.86) and six-lead (Leads 1, 2, 3, aVR, aVL, and aVF AUC 0.85) ECG readings [41]. Despite significant promise for these technologies, validation in the general population is ongoing, and they have yet to be evaluated in cancer patients.

## 4. Transthoracic Echocardiography

Baseline transthoracic echocardiography (TTE) is recommended for all patients prior to the commencement of antineoplastic therapy as part of cardiovascular risk stratification [5,12]. Subsequently, TTE remains the primary diagnostic tool for CTRCD, focusing on LVEF, global longitudinal strain (GLS), and diastolic function [7,8,9,47]. TTE, in addition, provides important structural information including valvular assessment and evaluation for the presence of pericardial effusions. Clinically, valvular heart disease is a known long-term complication of mediastinal radiation therapy [48,49]. Furthermore, pericardial effusion is a common incidental finding in cancer patients, either as a direct result of local cancer invasion or secondary to anti-neoplastic therapy, and is associated with poor clinical outcomes [50,51]. Without timely detection, abnormalities in cardiac function may progress to symptomatic HF, emphasizing the need for effective diagnostics. However, conventional echocardiography faces limitations due to inter-observer variability, cost and low sensitivity for initial subclinical changes. Integrating AI into echocardiographic analysis offers potential to improve diagnostic accuracy and sensitivity [52,53]. AI-driven approaches may enhance risk stratification and support individualized treatment strategies, advancing CTRCD management.

### 4.1. Left Ventricular Systolic Function

Guidelines recommend a baseline assessment of LV systolic function prior to the commencement of therapy [5,7,54]. Where possible, a three-dimensional (3D) measurement of LVEF and ventricular volumes are preferred, with superior inter-observer and temporal variability compared to two-dimensional (2D) measurements of estimations from other imaging modalities [55,56]. If 3D TTE is not available, the calculation of LVEF with 2D Simpson’s biplane method is the next recommended method. The administration of ultrasound enhancing contrast is suggested if inadequate image quality is achieved or if two or more contiguous segments are poorly visualized on 2D images [5,57].

**Figure 2:**
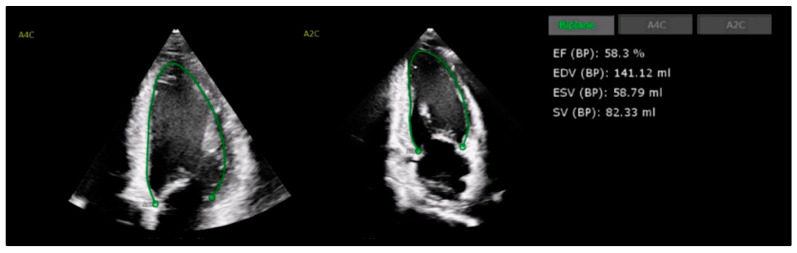
Example of AI model for automated measurement of left ventricular ejection fraction (LVEF), reported on apical 4-chamber (A4C) and apical 2-chamber (A2C) views. Model utilizes endocardial contour auto-generation to trace end-diastolic LV cavity volume (EDV), subsequently reporting LVEF by Simpson’s method.

In light of these recommendations, recent studies highlight AI’s potential to enhance LVEF assessment accuracy, expanding LV evaluation applications to healthcare providers beyond cardiology specialists, as shown in Figure 2. In a large-scale, blinded, randomized trial involving 3769 echocardiograms, AI-based LVEF evaluation improved compared with the cardiologists’ assessment by 10.4%, reducing significant variability to 16.8% compared to 27.2% in the sonographers’ measurements [58]. Furthermore, the automated AI calculation of LVEF utilizing hand-held ultrasound has also been shown to be accurate compared to traditional comprehensive TTE, offering the opportunity for greater access to LVEF measurements and surveillance [59,60].

In the oncology population, AI-enabled handheld ultrasound devices, utilizing AI-LVEF algorithms with real-time probe guidance, achieved sensitivity and specificity rates of 95% and 94% among minimally trained oncology staff—comparable to expert cardiologists [61]. In trastuzumab-treated patients with breast cancer, AI-derived LVEF measurements strongly correlated with the cardiologists’ evaluation (r = 0.895) [62]. These findings suggest AI–echocardiogram integration could reduce inter-observer variability, enhance diagnostic accuracy, and streamline clinical workflows.

Machine learning models have shown strong predictive capabilities for CTRCD via LV strain analysis as an early marker for LVSD. For example, circumferential strain changes in anterolateral and inferoseptal segments predicted LVEF decline in doxorubicin-treated patients, improving the AUC from 0.70 to 0.87 [63]. Stratification models have shown promise; in a large, multi-center cohort of older breast cancer patients (≥55 years), they achieved up to 73.55% accuracy in predicting early LVEF decline over a 12-month period by incorporating clinical and echocardiographic factors such as age, diabetes, hyperlipidemia, arrhythmia, and baseline LVEF, demonstrating the utility of multiple variables for risk stratification [64]. While these results are promising for the early detection of CTRCD, their validation in larger, diverse cohorts remains necessary.

### 4.2. Global Longitudinal Strain

A relative GLS decline >15% has emerged as an important metric of early LVSD, with utility for early cardiotoxicity detection; GLS has been shown to have sensitivity for subclinical myocardial dysfunction prior to LVEF decline [5,8,65]. However, GLS measurement can be associated with challenges with technical limitations, operator dependency, and inter-vendor variability; absolute GLS discrepancies up to 3.7% across ultrasound platforms have been described, and with −18% absolute GLS measurement being considered normal reference value, this variability in itself would exceed the 15% change that would be deemed clinically significant [66]. AI algorithms have been developed to address these issues by reducing observer bias and enhancing GLS measurement consistency.

**Figure 3:**
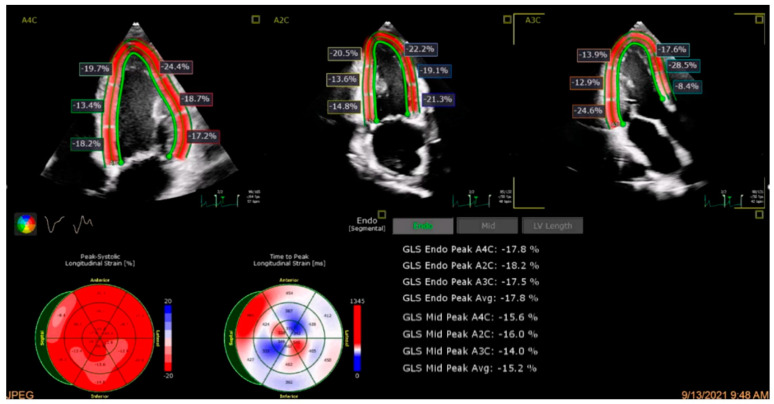
Example of artificial intelligence model to automate global longitudinal strain (GLS) on transthoracic echocardiogram. Through automated contouring of endocardial and epicardial borders on apical 4-chamber (A4C), apical 2-chamber (A2C), and apical 3-chamber (A3C) views, generation of peak endocardial and mid strain is presented.

AI-driven deep learning strain models have shown potential in standardizing GLS assessments by improving reproducibility and reducing inter-observer variability, an example of which is shown in Figure 3. A model using a dedicated software application achieved an intraclass correlation coefficient (ICC) of 0.81 (95% CI: 0.64–0.90) between novice and experienced clinicians compared to 0.62 (95% CI: 0.34–0.80) with conventional methods, supporting consistent evaluations across expertise levels [67]. Additionally, an AI model demonstrated enhanced predictive accuracy for HF detection in the PROMIS-HFpEF cohort and real-world datasets, achieving an AUC of 0.89, underscoring its potential to standardize GLS measurements across clinical settings [68].

AI shows promise in improving GLS accuracy for CTRCD monitoring. In doxorubicin-treated breast cancer patients, AI-enhanced GLS models increased prediction accuracy by 18%, raising the AUC from 0.70 to 0.87 through segmental strain analysis [63]. A DenseNet-121 model further refined GLS measurement accuracy, achieving an AUC of 0.84 and reducing inter-modality discrepancies with cardiac magnetic resonance by 15% [69]. However, in trastuzumab-treated patients, AI-automated GLS showed only a moderate correlation with manual measures (r = 0.541), with limitations in the apical three-chamber view [62]. A further investigation into technical constraints in AI echocardiography is needed.

A key consideration for AI in echocardiography is its potential to achieve sufficient diagnostic accuracy by integrating multiple variables. Multimodal AI models combining 2D and 3D LVEF, GLS, global circumferential strain (GCS), and biomarkers may offer a comprehensive approach to CTRCD detection. In a prospective study on HER2-positive breast cancer patients receiving anthracycline and trastuzumab therapy, a multimodal model achieved an AUC of 0.893 for CTRCD detection, with GCS alone identifying 50% of cases, outperforming individual metrics such as 3D LVEF (22%) and GLS (42%) [70]. These results imply that combining multiple echocardiographic parameters with biomarkers can significantly enhance diagnostic accuracy.

Despite advancements in AI for systolic dysfunction and GLS assessment, its application in evaluating diastolic dysfunction in CTRCD remains limited, indicating an area for future research. Studies have reported an odds ratio of 7.5 for cardiotoxicity within the first year of anthracycline therapy, with diastolic dysfunction manifesting on average 73 days before systolic decline in trastuzumab-treated patients, supporting guidelines recommending routine diastolic function monitoring [5,8,71,72]. AI-based methodologies show promise; an AI-assisted system achieved diagnostic accuracies of 0.90–0.92 for left ventricular diastolic function via multiview echocardiographic data without Doppler inputs, while a hybrid MMnet model achieved grading accuracies of 0.88–0.98 by integrating multimodal inputs [73,74].

**Figure 4:**
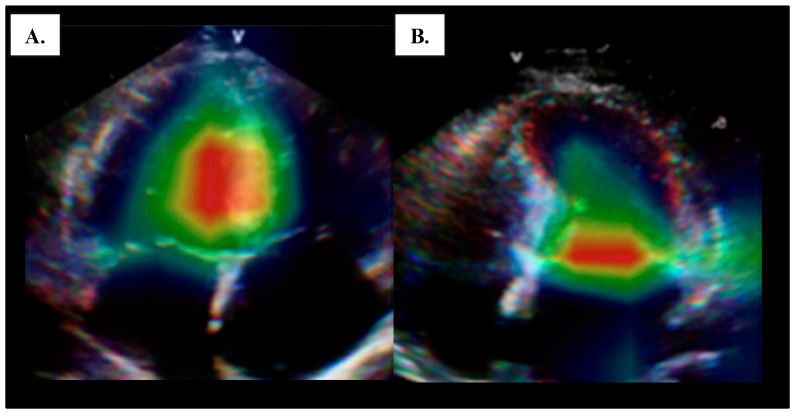
A machine learning model applied to a transthoracic echocardiographic 4-chmaber view to help differentiate constrictive pericardial disease from cardiac amyloidosis, which is the prototype for restrictive cardiac pathology; the GradCam images demonstrate focus of the AI model on the septum which has abnormal motion in constrictive pericarditis (panel (**A**)), and mitral annulus in cardiac amyloidosis (panel (**B**)), features which are important in the clinical differentiation of these entities. The generated heatmap depicts the focus of AI, whereby the red areas of those of highest focus. This figure is reproduced with permission [79].

Similarly, AI-driven echocardiography shows promise in detecting constrictive pericarditis (CP), potentially benefiting patients receiving radiation therapy. A 2023 ASCO analysis reported a 2.8-fold increased risk of CP in thoracic cancer patients undergoing radiation therapy, supported by MRI studies showing early radiation-induced cardiac fibrosis and remodeling—a risk noted by the American Heart Association [75,76,77]. Constriction is a challenging clinical entity to diagnose and results in heart failure years to decades after the initial radiation therapy applied to the chest. AI models employing speckle tracking and strain imaging have achieved high diagnostic accuracy for CP, with AUCs between 0.94 and 0.97, highlighting AI’s potential for early, precise CP diagnosis and improved management (Figure 4) [78,79,80].

Overall, AI-enhanced echocardiography demonstrates significant potential to advance CTRCD detection, improve risk stratification, and support personalized management in oncology. Early evidence suggests that AI enhances the accuracy and precision of LVEF and GLS measurements, with early work showing promise for predictive capabilities for other echocardiographic parameters associated with CTRCD, such as diastolic dysfunction and CP assessment. Future research would benefit from prioritizing large-scale validations and examining the utility of AI in these understudied areas to fully optimize its potential for improving cardiovascular outcomes.

## 5. Cardiac Computed Tomography, Cardiac Computed Tomography Angiography, and X-Ray

### 5.1. Cardiac CT

Given the overall burden of CAD in the community, along with the increased survivorship of cancer patients resulting in a longer lifespan with an increased risk of cardiovascular disease with increasing age, as well as the effect of accelerated CAD after radiation therapy applied to the chest or antineoplastic therapies that increase risk factors, screening for CAD is important in all oncology patients and survivors. Guidelines recommend baseline non-contrast cardiac CT for coronary artery calcium scoring (CAC) in patients with known risk factors for atherosclerotic CAD [3,5,12,81]. Clinically, this test holds importance as undetected subclinical coronary atherosclerosis may increase the risk of treatment-related cardiotoxicity and the overall risk of cardiovascular events [82,83]. In addition, in low-cardiovascular-risk patients, baseline CAC scoring is recommended in the setting of antineoplastic therapies with known vascular toxicities such as tyrosine kinase inhibitors like sorafenib and sunitinib, interferon-alpha and IL-2, or mediastinal radiation therapy [12,84,85].

In the oncology population, CAC scoring is a relatively accessible modality cardiovascular risk stratification and may be performed not only on dedicated cardiac CT scanning but also on non-gated chest CT scans for cancer surveillance or staging [86,87,88,89]. As such, many studies have explored the application of automated AI and machine learning models in calculating CAC, both to improve the quality and accuracy of coronary artery quantification and to reduce the time and cost burden [89,90,91]. For example, Lessmann et al. employed a multistage convolutional neural network model on 1744 chest CT scans of lung cancer patients, reporting an F_1_ precision and recall score of 0.84 and high inter-observer agreement [92].

Subsequently, Eng et al. trained a deep learning model to perform CAC scoring on non-gated CT scans with paired gated CT scans, reporting a sensitivity of 71–94% and positive predictive value of 88–91 for the detection of CAC scores ≥ 100 [93]. Zeleznik et al. evaluated 20,084 patients from the Framingham Heart Study and National Lung Screening Trial [94,95] (asymptomatic), PROMISE [96] (stable chest pain), and ROMICAT-II [97,98] (acute chest pain), reporting a significant correlation between an automated deep learning model for CAC scoring and manual quantification (Spearman’s coefficient 0.92, *p* < 0.0001) [91]. Additionally, their AI-CAC risk categorization was significantly correlated with cardiovascular mortality in patients with lung cancer through a median follow-up of 6.7 years.

### 5.2. CCTA

**Figure 5:**
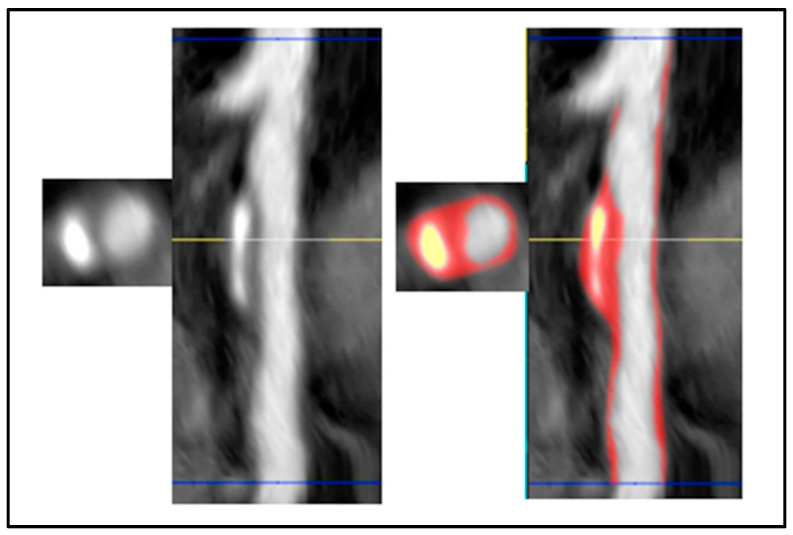
Artificial intelligence automation of vessel segmentation and plaque quantification in case of left anterior descending coronary artery stenosis, with noncalcified, soft, cholesterol rich plaque is denoted in red and calcified plaque in yellow. Figure reproduced with permission [103].

There has been an increasing shift towards the evaluation of acute and sub-acute chest pain with non-invasive cardiac CT angiography (CCTA) rather than invasive coronary angiography where possible, once acute ST-elevation myocardial infarction has been ruled out with ECG [88]. In the general population, multiple AI models have been evaluated to improve the accuracy and efficiency of CCTA to non-invasively quantify and localize coronary plaque burden, as well as assess for high-risk plaque features, as demonstrated in Figure 5 [99,100,101]. Zreik et al. evaluated the accuracy of AI on CCTA for the detection and characterization of coronary plaque (plaque or no plaque and calcified, non-calcified, or mixed plaque) and the degree of anatomically significant coronary stenosis (≥50% luminal narrowing) [101]. Utilizing a multi-task convolutional neural network model on 163 patients, they reported an accuracy of 0.77 for plaque detection and characterization and 0.80 for the detection of significant coronary stenosis. Another multi-national study by Coenen et al., involving 351 patients (Machine Learning Based CT Angiography Derived FFR: A Multi-Center Registry), applied a machine learning model to CCTA for a fractional flow reserve evaluation of coronary stenosis [102]. This model out-performed manual visual CCTA classification, with a diagnostic accuracy of 0.84 compared to 0.69 in the detection of hemodynamically significant disease.

Other studies have evaluated the utility of AI in assessing high-risk plaque features on CCTA, which include low attenuation plaque, spotty calcification and the napkin ring sign [100,104,105]. Early results from the Quantitative Coronary CT Angiography Evaluation for Evaluation of Clinical Outcomes (CONFIRM 2) study, which is ongoing, report on the ability of an AI-guided model to accurately and reproducibly quantify whole heart plaque burden, with promising results [106]. This study, conducted on 3551 patients across 13 countries, has also shown an association between a high AI-quantified CT score and increased clinical events including mortality and myocardial infarction [107].

In cancer patients, cardiac CT and CCTA predominantly play a role in the evaluation of potential cardiotoxicity with antineoplastic therapy, assisting in ruling out obstructive CAD in the presence of acute angina equivalent symptoms. For example, certain therapies such as the antimetabolites such as 5-FU are known to cause coronary vasospasm, which may mimic the presentation of acute coronary syndrome; CCTA can effectively rule out coronary atherosclerotic lesions in the support the diagnosis of 5-FU-related vasospasm in patients with supporting clinical context [12]. In addition, mediastinal radiation has been associated with diffuse coronary atherosclerosis that may present a decade or two after the initial radiation therapy, and it is often difficult to diagnose due to its atypical presentation and lack of localizing symptoms [84,108,109]. In radiation-induced heart disease, nuclear imaging may underestimate the degree of ischemia and CAD, creating a potential indication for coronary CT in this subpopulation; however, extensive calcification that may go hand in hand with radiation heart disease may also make CCTA technically challenging for the severity of stenosis quantification [85,108,110].

### 5.3. X-Ray

Another emerging avenue for cardiac evaluation in oncology patients is plain chest radiography, which has become an increasingly evaluated tool for AI modalities. Studies have shown the ability for deep learning models to quantify multiple cardiac parameters from a simple chest Xray, including left ventricular function, CAD, and valvular heart disease [111,112,113]. For cancer patients, this may hold promise as a non-invasive, low radiation screening tool for asymptomatic CTRCD.

Despite the potential applications and the promising ongoing research into AI models within this area, there is currently no literature directly assessing the role of AI-CCTA in cancer patients specifically, either for the evaluation of CAD burden prior to cancer therapy or for the evaluation of symptoms of potential cardiotoxicity. Future research may allow for the utilization of transfer learning models or novel AI tools to be applied directly to oncology patients, with the hope for improved diagnostic and prognostic accuracy and efficiency in both patients with cancer and cancer survivors.

## 6. Cardiac Magnetic Resonance Imaging

Cardiac magnetic resonance imaging (CMR) is considered the gold standard for assessing cardiac anatomy and function due to its high image resolution and superior tissue characterization [114]. Thus, CMR is widely recommended in clinical guidelines as the preferred imaging method for various indications, including the evaluation of masses, ischemia, cardiomyopathies, and assessing pericardial disease [114]. In cardio-oncology, CMR is the recommended modality for assessing cardiac masses and diagnosing myocarditis and for the assessment of LVSD in the setting of discordant LVEF measurements [12,115]. However, the widespread use of CMR in clinical practice has generally been limited by the time required for CMR and interpretation, the extensive training needed to develop expertise, overall cost and accessibility, and the presence of metal implants that may contraindicate CMR or degrade image quality [116].

As a result, automated CMR interpretation with machine learning techniques hold significant potential for improving the speed and accuracy of cardiovascular disease screening and diagnosis, through optimizing image acquisition, and enhancing image analysis and prognostication [16,117,118,119,120,121]. For example, Kustner et al. developed a deep learning generative adversarial network model on 3D CMR, utilizing super resolution (the retrospective motion-compensating reconstruction of low-resolution images) [122]. In 50 training patients and 16 validation patients, this study reported both a significant improvement in the spatial resolution of coronary vessel anatomy and reduced the scan time to less than 60 s when compared to standard high-resolution CMR imaging. Steeden et al. reported a similar technique for the acquisition of whole-heart imaging using a convolutional neural network applied to 500 CMRs [123]. With retrospective super resolution they demonstrated significantly increased image quality, including image sharpness, signal-to-noise ratio, and less image distortion compared to both the initial low-resolution images and conventional high-resolution images. The image acquisition time for low-resolution images was three times faster than that of high-resolution images, and there was no significant difference in vessel measurements of the great vessels between the super-resolution images and high-resolution images.

In addition to improved image acquisition efficacy, AI models have been trained to improve diagnostic accuracy with the recognition of subtle patterns and changes in myocardial structure and function, often beyond the detection capability of human interpretation [16]. Automated image segmentation allowing for the assessment of cardiac function, ventricular volumes, and myocardial mass has been a focus of multiple AI models [124,125]. Bai et al. demonstrated this using a convolutional neural network model on 4875 patients, noting an evaluative performance equal to that of expert manual measurements [126]. Another study of 210 cine CMR sequences assessed the utility of automated ventricular chamber segmentation with a deep learning model, noting good accuracy compared to expert manual assessments. Clinically, this model may allow for the accurate and automated evaluation of ventricular volumes [127].

### 6.1. Late Gadolinium Enhancement CMR

In the cardio-oncology population, the administration of gadolinium contrast allows for a more accurate evaluation of myocardial pathology such as fibrosis, scarring, or edema [124,128]. However, in addition to the contrast-associated risks, gadolinium-enhanced CMRs typically require longer scan times, are more taxing for patients, and require an experienced radiologist to manually review the images. To address this, AI models have attempted to further improve the assessment of myocardial tissue characteristics and overcome limitations in late gadolinium enhancement (LGE) quantification, including not only the logistical complexities but also gadolinium kinetics variability and inter-center differences in accuracy and reproducibility while analyzing T1 and T2 mapping data [16,118].

Studies have evaluated the utility of AI to improve automated landmark localization and segmentation and to improve the efficiency of LGE imaging [129,130]. Zhang et al. evaluated a convolutional neural network on virtual native-enhanced (VNE) images of 1348 patients with hypertrophic cardiomyopathy, reporting both significantly improved image quality compared to LGE images and good diagnostic correlation [131]. Furthermore, the processing of VNE images took less than one second from native T1 images and required no intravenous contrast.

### 6.2. CMR Strain Analysis

Myocardial strain analysis on CMR has been shown to allow for a sensitive quantitative assessment of cardiac function and may offer a tool for the earlier detection of myocardial dysfunction prior to an observed decline in LVEF [115,124]. The StrainNet study evaluated a convolutional neural network model for a strain analysis on cine CMR images of 161 healthy patients, reporting the superior performance of this model compared to the conventional assessment of global and segmental strain [132]. In a cohort of 223 patients with ischemic heart disease, another study found a similar performance of a deep learning synthetic strain model applied to cine images to that of subspecialty radiologists, with an ROC AUC of 0.90 for the detection of wall motion abnormalities [133]. These models may offer particular utility in the cardio-oncology population, as myocardial strain has been thought to precede clinical symptoms or decline in LVEF, particularly in the setting of anthracycline or trastuzumab therapies [134,135,136].

### 6.3. Myocarditis

Another entity that is important in the cardio-oncology practice is immune-checkpoint inhibitor-related myocarditis, which, although rare, carries a significant mortality and major adverse cardiac event burden and can be challenging to diagnose [137,138]. Although endomyocardial biopsy is the gold standard for diagnosis it is invasive and infrequently performed, and cardiac imaging with CMR is often a major diagnostic criterion [5]. A recent systematic review reported AI significantly improved the accuracy and efficiency of CMR in diagnosing myocarditis, providing potential prognostic value by enhancing the chances of successful outcomes by facilitating earlier diagnose and initiation of treatment [139].

Furthermore, AI-CMR models have been demonstrated to distinguish between myocarditis and other cardiac pathologies, overcoming challenges presented by such often presentations of myocarditis that may mimic other cardiac conditions. A prospective study assessed the diagnostic potential of texture analysis (TA) in heart failure-like myocarditis compared to endomyocardial biopsy (EMB) as the reference standard [140]. Participants from the CMR in Myocarditis (MyoRacer) trial underwent biventricular EMB and CMR at 1.5 T with TA application on T1 and T2 mapping, showing that TA applied to CMR T1 and T2 mapping provided superior diagnostic accuracy for acute and chronic-like myocarditis compared to traditional Lake Louise criteria or mean T1/T2 values.

### 6.4. Cardiac Amyloidosis

CMR also plays a role in the non-invasive evaluation and diagnosis of cardiac amyloidosis, typically seen as diffuse or patchy LGE uptake throughout the myocardium [15]. As such, several studies have evaluated AI modeling for the improved diagnostic accuracy of cardiac amyloidosis on CMR. One study applied a convolutional neural network model to 502 patients (82 with endomyocardial biopsy-confirmed cardiac amyloidosis), reporting a diagnostic AUC accuracy of 0.96 with a fine-tuning deep learning technique [141]. More recently, a single-center study by Eckstein et al. evaluated 43 patients with confirmed cardiac amyloidosis, collating a 41-feature matrix decision tree based on CMR cardiac function and multi-chamber strain. This supervised machine learning model had an AUC accuracy of 0.96 and sensitivity of 94% for diagnosis [142].

Although promising, the application of AI-driven CMR in clinical practice, especially in cardio-oncology, still faces several challenges, including limitations to training and the validation of algorithms due to scarce datasets, variability in CMR acquisition protocols, and differences in interpretation software, all of which affect the generalizability of AI models. Additionally, regulatory approval and clinical integration are still lacking. Future advances should focus on overcoming sample size limitations through database sharing across institutions, developing standardized validation algorithms to compare different machine learning techniques to expert interpretations for CMR datasets, and establishing the effectiveness of these models in clinical practice, allowing for real-time decision making in managing cardiotoxicity in cancer patients.

## 7. Nuclear Imaging

In recent years, there have been substantial advancements in both the underlying technology and clinical utility of AI models applied to nuclear imaging modalities [143]. Specific to cardiac nuclear medicine, modalities include positron emission tomography (PET), single-positron emission computed tomography (SPECT), and multigated acquisition scan (MUGA). These nuclear imaging techniques are primarily used for the assessment of cardiac function and myocardial perfusion [3,12]. Although less widely utilized, currently, due to radiation exposure and alternative imaging modalities, MUGA may be used for the assessment of cardiac function where TTE and CMR are not available or appropriate, with highly reproducible measurements of LVEF [2,5].

### 7.1. PET

Clinically, cardiac PET imaging remains the recommended modality for the evaluation of microvascular perfusion and myocardial metabolism, with superior temporal resolution to other modalities [2,144]. Often combined with CT for anatomical localization, PET has also been shown to not only accurately quantify myocardial ischemia but to independently predict adverse clinical events [145]. Similar to CMR, multiple deep learning models have been evaluated for improved PET efficiency, including improvements in image quality and acquisition, focusing on reducing image noise and improving super resolution [143,146]. In 2019, Wang et al. proposed an artificial neural network model to improve the quantification of myocardial perfusion on PET imaging, reporting a significantly improved detection of both transmural and non-transmural perfusion defects [147]. Another study evaluated a deep learning model to reconstruct and denoise low-dose coronary PET images, demonstrating a similar signal-to-noise ratio compared to that of high-dose PET images, with one tenth of the scan time [148]. In a study of 166 patients, Ladefoged et al. applied a deep learning U-net model to cardiac fluorodeoxyglucose (FDG)-PET scans, noting similar diagnostic accuracy with their model with both a 1% and 10% FDG dose reduction compared to full-dose scans [149].

In addition to software advancements, AI developments in PET technology such as the addition of new cameras and probes for the rapid detection of substrates and radioisotopes offer significant enhancements in detecting early-stage heart diseases [150,151]. Furthermore, FDG-PET imaging is recommended for the evaluation of metastatic malignancies and intracardiac masses and for the assessment of myocarditis if CMR is unavailable or contraindicated [5,12]. Studies have demonstrated the utility of AI in whole-body FDG PET for diagnostic accuracy, showing potential comparative to expert opinion [151,152]. Further, it has been postulated that machine learning models may be trained to detect changes in FGD uptake over time, tracking the progression of distribution or the efficacy of anti-neoplastic therapy [1,11]. AI models have also been developed to estimate the coronary calcium score based on a non-gated CT chest, which may be applied to scout images on FDG-PET used for the staging of cancer to both determine the presence of CAD and estimate the degree of burden by opportunistic screening [153,154]. These represent only a few examples of the myriad literature in this field, utilizing different AI techniques to improve PET image quality and efficiency [143,146,155].

### 7.2. SPECT

SPECT myocardial perfusion imaging is a very widely utilized non-invasive modality for the evaluation of CAD. In this area, multiple AI models have been shown to be superior to manual visual assessments in the evaluation and quantification of ischemia related to CAD, as well as the prediction for the need of revascularization [156,157,158]. Similar to PET, AI models have also been evaluated for improved SPECT image quality, faster acquisition and processing time, and improved diagnostic accuracy [118,159,160,161,162]. Arsanjani et al. reported the similar accuracy of a fully automated model in the quantification of CAD compared to an expert visual assessment in 995 patients [156]. This group subsequently demonstrated the accuracy of ML on SPECT perfusion images to predict the risk of coronary revascularization in 713 patients compared to expert clinical consensus [158].

More recently, a study by Betancur et al. evaluated a deep convolutional neural network model in 1638 patients without known CAD, reporting an AUC accuracy of 0.80 and sensitivity of 82.3% for the prediction of obstructive CAD [163]. This group also reported the superior predictive value of this machine learning SPECT imaging model compared to a physician’s diagnosis to predict major adverse cardiac event outcomes in 2619 patients over a mean follow-up of 3.2 years [164].

In oncology patients, nuclear imaging holds its main role in the evaluation of myocardial perfusion, either as a baseline assessment of CAD prior to chemotherapy in high-risk patients or as evaluation and surveillance of low-risk patients with suspected CAD following the commencement of therapy [3,144]. PET has specific utility in the assessment of CAD after direct mediastinal radiation [165,166]. Overall, although these models may offer substantial improvements in the diagnostic and prognostic potential of nuclear imaging, with an improved image quality and reduced scan time and contrast requirement, most models are yet to be validated on large external cohorts. Furthermore, there is a paucity of data regarding the direct application of these models in oncology patients, specifically in the setting of potential CTRCD [2].

## 8. Discussion 

In oncology patients, improvements in long-term survivorship and the ongoing development of novel antineoplastic therapies have led to an increased burden of cardiovascular disease and complications including cardiotoxicity. As such, there is an increased reliance on cardiac imaging to allow for the early detection and characterization of cardiovascular disease or cancer therapy-related cardiac dysfunction.

AI is a rapidly evolving field across all areas of clinical medicine and, in particular, in the accuracy and efficiency of medical imaging. Within cardiac investigations, AI-ECG has been shown to have substantial potential as an accessible tool for the diagnosis and prognosis of many conditions, including and specifically relevant to CTRCD. Several AI-ECG applications are presently accessible for the routine assessment of cancer patients, including the evaluation of LVSD, arrythmia, and cardiac amyloidosis. With the ongoing development of wearable technology and the progressive validation of AI models on single-lead ECG readings, this may offer enormous clinical significance for oncology patients.

Echocardiography remains the cornerstone of cardiac evaluation for the diagnosis of CTRCD, manifested as LVSD. The automated calculation of LVEF, along with machine learning models for the enhancement of GLS, have already been shown to add significant diagnostic and prognostic value in both the general and oncology population. The further specific evaluation and development of AI modalities for disease characterization will likely be the next frontier for cancer patients. Furthermore, the progression of hand-held ultrasound may allow for a broader spectrum of utility in this setting, with potential for screening in the oncology setting.

Within cardiac CT, AI-driven models for the quantification and localization of atherosclerotic plaque burden have previously been shown to increase the clinical utility of these tools. This holds particular relevance in the oncology population, whereby non-contrast cancer screening CT scans may allow for baseline risk stratification without further invasive investigation or contrast load. Furthermore, large cohort studies have shown the diagnostic accuracy of AI-enhanced CCTA for the characterization of coronary atherosclerosis, offering a non-invasive investigation for angina-equivalent symptoms in cancer patients.

Advancements in both CMR and cardiac nuclear imaging have focused on image acquisition and reconstruction, automated qualitative assessments, reduced scan time and contrast requirement. In these modalities, AI has shown substantial promise in increasing utility in many clinical settings, with improved diagnostic and prognostic potential. Currently, larger-volume studies, specifically those evaluating the cardio-oncology population, are ongoing for the validation of these methodologies.

## 9. Conclusions

Overall, although there have been substantial developments in the role of AI in the fields of ECG, electronic medical records and cardiac imaging, there remain myriad opportunities for its expanded utility for cardio-oncology patients. There is an ongoing role for collaborative relationships between clinicians and AI bioengineers in further developing models that can help clinicians and may ultimately improve patient outcomes and decrease cardiovascular sequalae, particularly associated with CTRCD. At present, AI applied to testing as reviewed herein improves efficiency and reproducibility of results and acts as a marker for increased disease risk with a view to further targeted evaluation, and is not diagnostic in itself.
